# Impact of acute exercise on brachial artery flow-mediated dilatation in young healthy people

**DOI:** 10.1186/1476-7120-10-39

**Published:** 2012-10-02

**Authors:** In-Chang Hwang, Kyung-Hee Kim, Won-Suk Choi, Hyun-Jin Kim, Moon-Sun Im, Yong-Jin Kim, Sang-Hyun Kim, Myung-A Kim, Dae-Won Sohn, Joo-Hee Zo

**Affiliations:** 1 Division of Cardiology, Department of Internal Medicine, Seoul National University College of Medicine, Seoul, Korea; 2 Division of Cardiology, Department of Internal Medicine, Seoul National University Boramae Medical Center/Seoul National University College of Medicine, Seoul, Korea

**Keywords:** Flow-mediated dilatation, FMD, Acute exercise

## Abstract

**Background:**

Although chronic effects of exercise on endothelial function are established, the impact of acute exercise on flow-mediated dilatation (FMD) of brachial artery has not been elucidated yet.

**Methods:**

Eighty-six young healthy volunteers were prospectively enrolled from January 2011 to December 2011. The subjects completed FMD tests at rest and immediately after treadmill exercise test. Primary outcome was the impact of acute exercise on FMD, measured by the difference of FMD before and after exercise. Secondary outcomes were the relationship of gender and exercise habit with FMD.

**Results:**

Seventy-four subjects who met the eligibility criteria were included for analysis. Thirty-five (47.3%) were male, and the mean age was 22.7±2.7 years. FMD was reduced after exercise (8.98±4.69 to 7.51±4.03%; *P*=0.017) and the reduction was found in female group (10.36±5.26 to 7.62±3.71%; *P*=0.002) but not in male group. Post-exercise FMD was significantly impaired in subjects who did not exercise regularly (6.92±3.13% versus 8.95±5.33%; *P*=0.003). The decrease of FMD after exercise was greater in female group (−2.75±5.28% versus 0.27±3.24%; *P*=0.003) and was associated with exercise habit (β=2.532; *P*=0.027).

**Conclusions:**

In healthy young subjects, FMD was reduced after a bout of acute exercise. The impact of acute exercise showed significant differences according to gender and exercise habit. FMD impairment after acute exercise was observed in females and subjects without regular exercise.

## Background

Endothelial dysfunction is an important factor in the development of atherosclerosis, hypertension, and coronary artery disease (CAD) [1-4]. Impaired endothelial function is observed in subjects with risk factors for atherosclerosis and in patients with essential hypertension [2,5,6]. Also it is an important predictor of long-term cardiovascular events [7,8].

There are several methods to assess endothelial dysfunction and flow-mediated dilatation (FMD) is the most widely used non-invasive method to assess the endothelial function, reflecting the endothelial nitric oxide (NO) dependent vasodilation in response to occlusion-induced reactive hyperemia [5,9]. As FMD is non-invasive and reliable, allows repeated measurements [10,11], and also provides independent prognostic information of future cardiovascular events, the importance of FMD in clinical and research fields is growing [7,12,13].

For decades, various physiologic characteristics of FMD have been investigated. Aging and smoking are associated with reduced FMD [6,14,15]. Also, there are significant differences of FMD according to gender. Celermajer et al. reported that the pattern of age-related decline in endothelial function is different according to gender, and the progressive endothelial dysfunction appeared to occur earlier in men than in women [16]. Pahkala et al. reported that physical activity improved endothelial function in male adolescents, while female subjects did not show association [17]. Exercise training, which reduces cardiovascular risk, has been found to preserve endothelial function and improve FMD [17-21]. Despite the abundance of evidence regarding the chronic effect of exercise on FMD, the impact of acute exercise is still inconclusive. Several researchers have reported reduction of FMD immediately after exercise [22-24], while others have reported improvement [25,26]. Utilization of acute exercise model is advantageous for prescription of appropriate exercise protocol for patients, experimental control of confounding variables, and investigation of mechanisms of acute exercise response [27].

Given that a better comprehension of acute exercise model is beneficial in clinical and research fields, it is important to investigate the impact of acute exercise on FMD. We investigated the change of FMD after a bout of acute exercise and the influence of gender and exercise habit on FMD.

## Methods

### Study population

Eighty-six young healthy volunteers aged from 20 to 29 were recruited from January 2011 to December 2011. Subjects were excluded if they have established cardiovascular disease, hypertension, diabetes mellitus (DM), hypercholesterolemia, chronic kidney disease and pulmonary disease. Subjects with orthopedic conditions that limit exercise were also excluded from recruitment. We also excluded those who were smoking, considering the possible influence from it. All the volunteers were recruited from community.

The study was approved by the Institute of Review Board of Seoul National University Boramae Medical Center.

### Study design and data collection

After measurement of baseline FMD, participants performed treadmill protocol and post-exercise FMD measurement was followed. All measurements were performed following a 6-hour fast and an 8-hour abstinence from caffeine and/or alcohol and at least 24 hour after strenuous physical activity. Before measuring baseline brachial artery FMD, blood sampling was done for lipid profile. Then baseline brachial artery FMD was measured after a resting period of at least 20 minutes. All measures were performed under standardized conditions in a quiet, temperature-controlled room. Participants performed standard Bruce multistage maximal treadmill protocol until exhaustive point, and brachial artery FMD was measured again after the heart rate returned to baseline level.

Baseline demographic data on gender, age, height, weight, body mass index (BMI, kg/m^2^), smoking status, exercise habit, and family history of hypertension, DM, hypercholesterolemia, and CAD was collected at the time of recruitment. Exercise habits were defined as following 2 categories; subjects who exercise for 3 or more days a week at 30 minutes per session, and others who exercise less than 3 days a week or irregularly.

Primary outcome was the impact of acute exercise on FMD, measured by the difference of FMD before and after exercise. Secondary outcomes were the relationships of gender, smoking status and exercise habit with FMD.

### Flow-mediated dilatation

**Figure 1:**
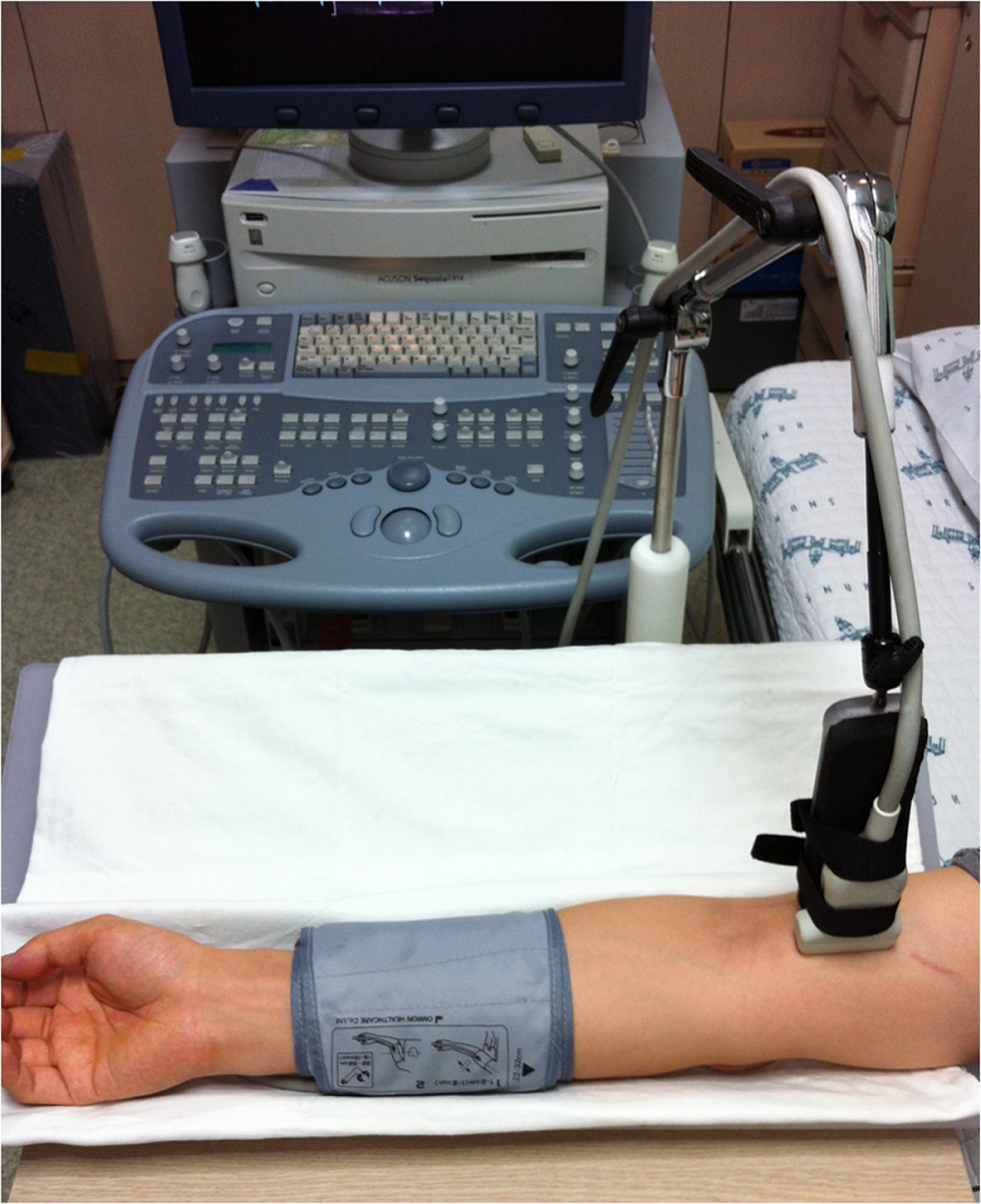
**Experimental setup for measurement of FMD.** Participants were rested supine with the right arm extended and immobilized. Pneumatic cuff was placed distal to the olecranon process. A 7.5 or 10 MHz multifrequency linear array probe was positioned in the distal third of the upper arm and custom-made probe-holder was used for stable transducer position during FMD measurement.

Experimental setup for measurement of FMD was shown in Figure 1. Participants rested supine with the right arm extended and immobilized with foam, supported at an angle of about 80 degree from the torso. Heart rate, systolic and diastolic blood pressures were determined from an automated sphygmomanometer on the contralateral arm. For the assessment of FMD response, a rapid inflation/deflation pneumatic cuff was positioned on the imaged arm distal to the olecranon process to provide a stimulus to forearm ischemia. A 7.5 or 10 MHz multifrequency linear array probe attached to a high-resolution ultrasound machine was used to image the brachial artery in the distal third of the upper arm. Ultrasound parameters were set to optimize longitudinal, B-mode images of the lumen/arterial wall interface. After a resting period of at least 20 minutes, 1 minute of baseline recording of the brachial artery diameter was performed. Subsequently, the occlusion cuff was inflated to >200 mmHg for 5 minutes. Brachial artery diameter recording was restarted at least 30 seconds before cuff deflation and continued for 3 minutes thereafter. Peak artery diameter and the time to reach this peak after cuff deflation were recorded. Electrocardiogram gaiting was utilized to capture end-diastolic brachial artery diameter, and post-test analysis was performed manually. FMD was calculated as the percent rise of peak diameter from the preceding baseline diameter and measured at every 1 minute after deflation for 3 minutes. Reliability analyses showed excellent values of interclass correlation coefficient between brachial artery diameters at each time point.

### Treadmill exercise

Following pre-exercise FMD measurement, treadmill exercise was performed following the standard Bruce protocol. If participants achieved the age-predicted maximum heart rate; requested termination of the exercise; developed severe chest pain, fatigue, leg discomfort or dyspnea; developed frequent premature ventricular beats; developed systolic blood pressure >250 mmHg or a drop in pre-test systolic blood pressure >10 mmHg; or developed any other problems, the exercise was terminated. When a subject was unable to reach at least 85% of age-predicted maximum heart rate, the level of exercise performed was considered inadequate, and the result was excluded from data analysis.

### Statistical analysis

Categorical variables are presented as frequencies and percentages, and continuous variables as means ± standard deviations (SD) or medians with interquartile ranges (IQR; 25th – 75th percentiles). For variables showing skewed distributions, logarithmic transformation was done. Group comparisons were performed with student *t* test or paired *t* test. The χ^2^ test or the Fisher’s exact test was used for categorical variables. Interclass correlation coefficient was used for reliability analysis. To evaluate the correlation between variables, Pearson’s correlation coefficient was used. Linear regression analyses were used to examine the determinants of FMD before and after exercise. Multiple linear regression analysis was used on all variables of *P* values <0.20 by univariate analyses. Analysis of covariance (ANCOVA) and repeated measure analysis of covariance (RM-ANCOVA) were performed to examine differences between FMD before and after exercise, using pre-exercise baseline brachial artery diameters as covariance. All of the statistical analyses were performed using SPSS 18.0 (SPSS Inc, Chicago, Ill) and a *P* value <0.05 was considered statistically significant.

## Results

### Subject characteristics

Among 86 subjects recruited, 12 subjects were excluded from data analyses because of incomplete data, inadequate treadmill exercise or current smoking and 74 subjects were included in data analyses. Subject characteristics and the differences between genders are presented in Table 1. The subjects of this study were young men (n = 35; 47.3%) and women (n = 39; 52.7%) with a mean age of 22.7 ± 2.7 years. Subjects who exercise regularly (3 or more days a week) were 19 (54.3%) in male group and 8 (20.5%) in female group, with significant difference between genders (*P* = 0.004). Regarding lipid profile of subjects, serum level of high-density lipoprotein-cholesterol (HDL-C) was higher in female group (61.0 [55.0 – 70.0] versus 52.0 [46.0 – 58.0] mg/dl; *P* = 0.004) while other profiles did not show differences. There were no significant differences in family history of cardiovascular disease, hypertension, DM, and hypercholesterolemia.

**Table 1:** Subject characteristics

	**Total subjects (N = 74)**	**Male (N = 35)**	**Female (N = 39)**	***P*value**
Age (year)	22.76 ± 2.71	22.26 ± 2.34	23.21 ± 2.96	0.134
Height (cm)	168.15 ± 7.56	173.76 ± 5.88	163.11 ± 4.88	<0.001
Weight (kg)	60.70 ± 9.58	67.43 ± 8.29	54.65 ± 5.96	<0.001
BMI (kg/m^2^)	21.36 ± 2.15	22.29 ± 2.11	20.51 ± 1.83	<0.001
Family history				
- FHx of Hypertension	19 (25.7%)	8 (22.9%)	11 (28.2%)	0.790
- FHx of DM	4 (5.4%)	2 (5.7%)	2 (5.1%)	1.000
- FHx of Dyslipidemia	2 (2.7%)	1 (2.9%)	1 (2.6%)	1.000
- FHx of CAD	2 (2.7%)	1 (2.9%)	1 (2.6%)	1.000
Exercise (3 or more days/week)	27 (36.5%)	19 (54.3%)	8 (20.5%)	0.004
Laboratory tests				
Cholesterol (mg/dl)*	156.0 (140.5 – 179.5)	151.0 (131.3 – 177.0)	157.0 (149.0 – 181.0)	0.146
Triglyceride (mg/dl)*	64.0 (45.0 – 81.5)	69.0 (42.8 – 92.3)	57.0 (45.0 – 77.0)	0.170
HDL-C (mg/dl)*	56.0 (50.0 – 63.0)	52.5 (47.8 – 59.3)	61.0 (55.0 – 70.0)	0.003
LDL-C (mg/dl)*	87.0 (73.5 – 107.0)	86.0 (64.8 – 110.5)	87.0 (76.0 – 103.0)	0.782

Values are mean ± SD, median with IQR (25th – 75th percentiles), or absolute number (%).

* Logarithmic transformation was done for these variables due to skewed distribution.

BMI = body mass index; CAD = coronary artery disease; DM = diabetes mellitus; FHx = family history; HDL-C = high-density lipoprotein-cholesterol; IQR = interquartile range; LDL-C = low-density lipoprotein-cholesterol.

### FMD measurements

Brachial artery structural and functional data are summarized in Table 2. Both pre-exercise and post-exercise baseline brachial artery diameters were higher in male group. Pre-exercise FMD was lower in male group, but post-exercise FMD showed no significant differences. The mean of post-exercise FMD was lower than pre-exercise FMD, and the difference (ΔFMD, [post-exercise FMD, %] – [pre-exercise FMD, %]) was greater in female group.

**Table 2:** Brachial artery data at rest and after exercise

	**Total subjects (N = 74)**	**Male (N = 35)**	**Female (N = 39)**	***P*value**
Baseline SBP (mmHg)	113.09 ± 9.43	116.74 ± 8.68	109.82 ± 8.95	0.001
Baseline DBP (mmHg)	73.15 ± 5.95	75.03 ± 6.55	71.46 ± 4.85	0.011
Baseline HR (bpm)	73.16 ± 8.23	71.89 ± 8.10	74.31 ± 8.28	0.208
Pre-exercise				
Baseline diameter (mm)	3.32 ± 0.42	3.56 ± 0.37	3.11 ± 0.36	<0.001
FMD (%)	8.98 ± 4.69	7.44 ± 3.41	10.36 ± 5.26	0.007
Post-exercise				
Baseline diameter (mm)	3.51 ± 0.41	3.71 ± 0.37	3.32 ± 0.35	<0.001
FMD (%)	7.51 ± 4.03	7.39 ± 4.42	7.62 ± 3.71	0.807
ΔFMD (%)†	−1.47 ± 4.54	−0.05 ± 3.04	−2.75 ± 5.28	0.008
Treadmill exercise				
Exercise duration (sec)*	665 (613 – 747)	735 (658 – 823)	620 (600 – 700)	<0.001
METS*	13.3 (12.1 – 14.3)	13.7 (13.1 – 15.8)	12.4 (11.7 – 13.4)	0.001
Maximum HR (%)‡	90.23 ± 1.96	90.07 ± 1.80	90.41 ± 2.12	0.377

Values are mean ± SD, median with IQR (25th – 75th percentiles), or absolute number (%).

* Logarithmic transformation was done for these variables due to skewed distribution.

† ΔFMD = post-exercise FMD (%) – pre-exercise FMD (%).

‡ Maximum HR (%) = percentage of age-predicted maximum heart rate.

BPM = beat per minute; DBP = diastolic blood pressure; HR = heart rate; IQR = interquartile range; METS = metabolic equivalents; SBP = systolic blood pressure.

**Figure 2:**
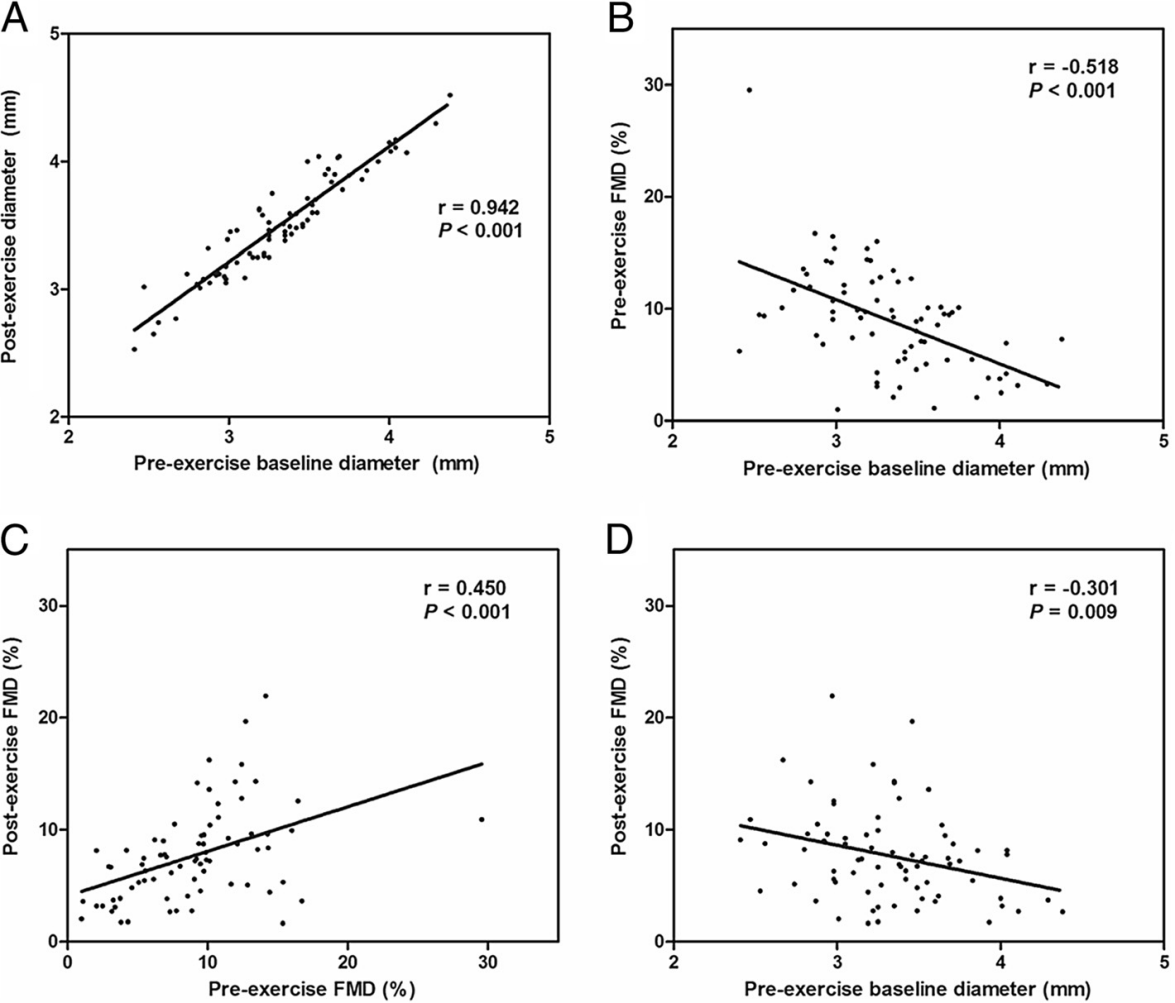
**Correlation analyses of brachial artery diameters and FMD measurements.** (**A**) Pre- and post-exercise brachial artery diameters were significantly correlated (r = 0.942; *P* <0.001). (**B**) Pre-exercise diameter and pre-exercise FMD showed significant correlation (r = −0.518; *P* <0.001). (**C**) Pre-exercise FMD and post-exercise FMD (r = 0.450; *P* <0.001), and (**D**) pre-exercise diameter and post-exercise FMD (r = −0.301; *P* = 0.009) were also significantly correlated. FMD = flow-mediated dilatation.

The correlation of baseline brachial artery diameters and FMD were assessed (Figure 2). Pre-exercise baseline brachial artery diameter was closely correlated with post-exercise diameter (r = 0.942; *P* <0.001) (Figure 2A). Also, there were significant correlations between pre-exercise diameter and pre-exercise FMD (r = −0.518; *P* <0.001) (Figure 2B), pre-exercise FMD and post-exercise FMD (r = 0.450; *P* <0.001) (Figure 2C), and pre-exercise diameter and post-exercise FMD (r = −0.301; *P* = 0.009) (Figure 2D). Baseline brachial artery diameters showed significant increase after exercise. The mean of pre-exercise brachial artery diameter was 3.32 ± 0.42 mm and post-exercise was 3.51 ± 0.41 mm (*P* <0.001 by paired *t* test).

### Association of the burden of exercise with FMD

**Figure 3:**
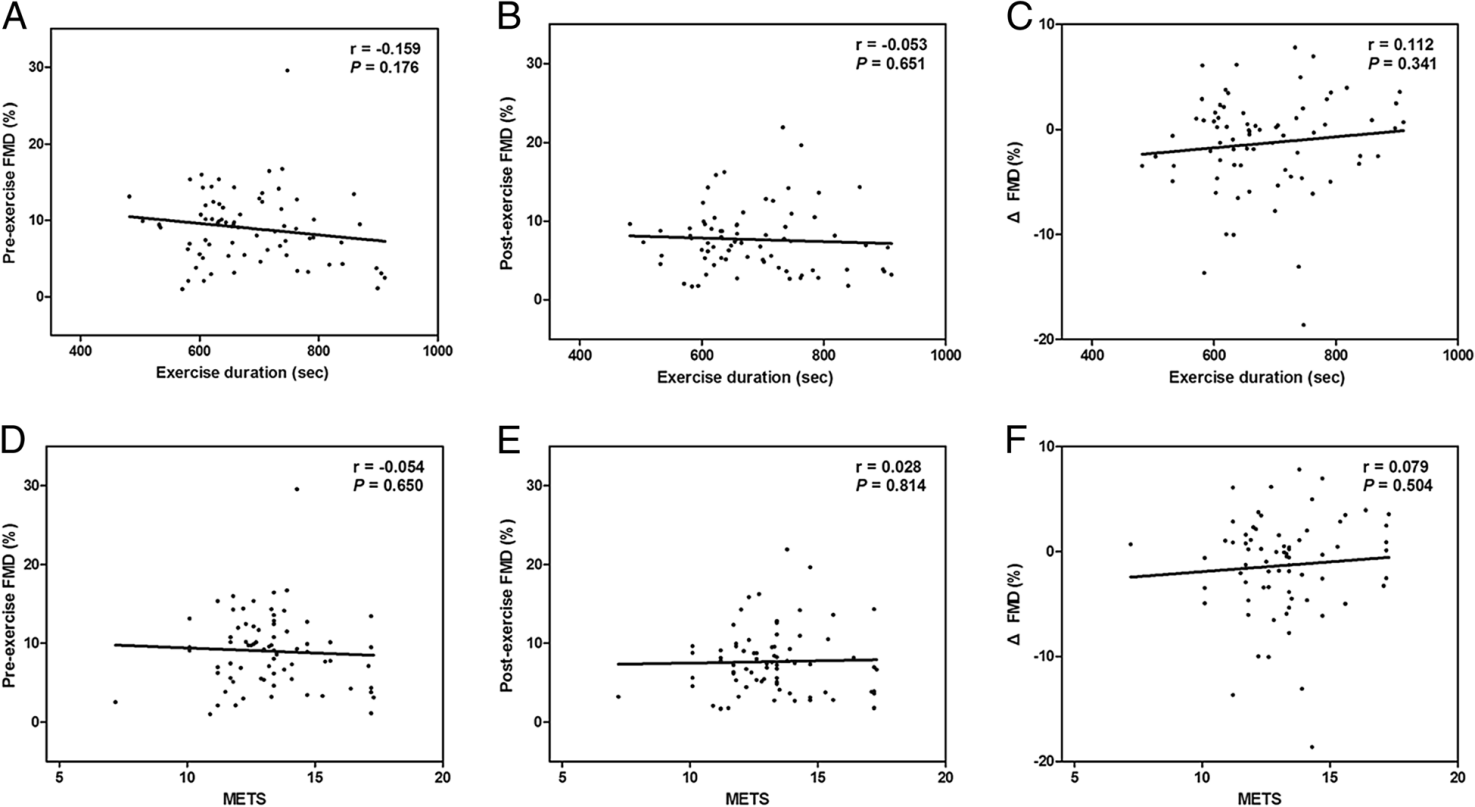
**Association of the burden of exercise with FMD.** Burden of exercise was measured as exercise duration and METS. None of the exercise parameters were associated with FMD measurements; (**A**) exercise duration with pre-exercise FMD, (**B**) exercise duration with post-exercise FMD, (**C**) exercise duration with ΔFMD, (**D**) METS with pre-exercise FMD, (**E**) METS with post-exercise FMD, and (**F**) METS with ΔFMD. FMD = flow-mediated dilatation; METS = metabolic equivalents.

Using Pearson’s correlation coefficient, the associations of pre-exercise FMD, post-exercise FMD and ΔFMD with the burden of acute exercise were analyzed (Figure 3). All of the FMD measurements were not associated with the burden of exercise, measured as exercise duration and metabolic equivalents (METS) during treadmill exercise test.

### Determinants of pre-exercise and post-exercise FMD

We evaluated determinant factors of pre-exercise FMD, post-exercise FMD and the difference of them (Table 3). By multiple linear regression analyses, pre-exercise FMD showed marginal significance with male gender (β = −2.036; 95% confidence interval [CI] -4.362 to 0.289; *P* = 0.085). Post-exercise FMD was preserved in subjects who exercise 3 or more days a week (β = 2.064; 95% CI 0.133 to 3.995; *P* = 0.037). The difference between FMD at rest and after exercise showed similar results. ΔFMD was significantly associated with exercise habit (β = 2.532; 95% CI 0.300 to 4.765; *P* = 0.027).

**Table 3:** Determinants of pre- and post-exercise FMD, and ΔFMD

**Variables**	**Univariate analysis**			**Multivariate analysis**		
	**β**	**95% CI**	***P*value**	**β**	**95% CI**	***P*value**
**Pre-exercise FMD**						
Age (year)	0.045	−0.361 – 0.451	0.826			
Male	−2.923	−5.004 - -0.843	0.007	−2.036	−4.362 – 0.289	0.085
Exercise (3/week)	−0.979	−3.239 – 1.282	0.391			
BMI (kg/m^2^)	−0.589	−1.082 - -0.096	0.020	−0.368	−0.900 – 0.165	0.173
Cholesterol (mg/dl)*	8.928	−2.818 – 20.674	0.134	5.596	−5.974 – 17.167	0.338
Triglyceride (mg/dl)*	2.098	−3.229 – 7.426	0.435			
HDL-C (mg/dl)*	6.088	−6.538 – 18.715	0.340			
LDL-C (mg/dl)*	2.757	−5.465 – 10.980	0.506			
**Post-exercise FMD**						
Age (year)	−0.145	−0.493 – 0.203	0.409			
Male	−0.231	−2.115 – 1.653	0.807			
Exercise (3/week)	1.603	−0.315 – 3.521	0.100	2.064	0.133 – 3.995	0.037
BMI (kg/m^2^)	−0.046	−0.487 – 0.394	0.835			
Exercise duration (sec)*	−5.219	−20.414 – 9.976	0.496			
METS*	2.031	−12.282 – 16.345	0.778			
Cholesterol (mg/dl)*	11.230	1.310 – 21.150	0.027	9.985	−6.383 – 26.353	0.228
Triglyceride (mg/dl)*	0.254	−4.349 – 4.856	0.913			
HDL-C (mg/dl)*	−0.704	−11.634 – 10.226	0.898			
LDL-C (mg/dl)*	6.355	−0.582 – 13.291	0.072	1.164	−10.166 – 12.493	0.838
**ΔFMD (%)**						
Age (year)	−0.190	−0.582 – 0.201	0.336			
Male	2.692	0.665 – 4.719	0.010	1.214	−1.147 – 3.574	0.309
Exercise (3/week)	2.582	0.465 – 4.699	0.018	2.532	0.300 – 4.765	0.027
BMI (kg/m^2^)	0.543	0.063 – 1.023	0.027	0.425	−0.093 – 0.942	0.106
Exercise duration (sec)*	5.354	−11.775 – 22.483	0.535			
METS*	3.072	−13.048 – 19.192	0.705			
Cholesterol (mg/dl)*	2.302	−9.251 – 13.854	0.692			
Triglyceride (mg/dl)*	−1.845	−7.012 – 3.323	0.479			
HDL-C (mg/dl)*	−6.793	−19.005 – 5.420	0.271			
LDL-C (mg/dl)*	3.598	−4.352 – 11.547	0.370			

* Logarithmic transformation was done for these variables due to skewed distribution.

BMI = body mass index; CI = confidence interval; FMD = flow-mediated dilatation; HDL-C = high-density lipoprotein-cholesterol; LDL-C = low-density lipoprotein-cholesterol; METS = metabolic equivalents.

### Impact of acute exercise on FMD

**Figure 4:**
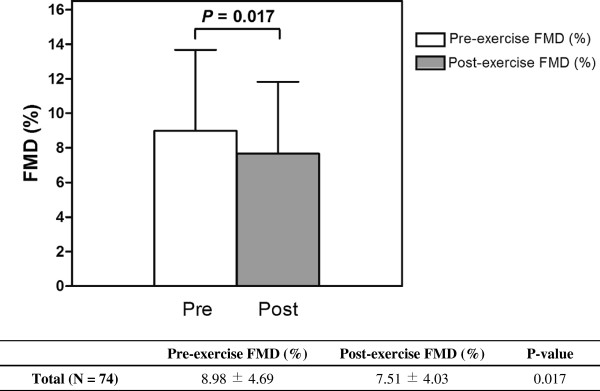
**Difference of FMD between pre-exercise and post-exercise.** Values are mean ± standard deviation. *P* values were calculated by paired *t* test. FMD was significantly reduced after a bout of acute exercise. FMD = flow-mediated dilatation.

Primary outcome of this study was the impact of acute exercise on FMD in healthy young people, and FMD was significantly reduced after exercise (8.98 ± 4.69 versus 7.51 ± 4.03%; *P* = 0.017 by paired *t* test) (Figure 4).

**Figure 5:**
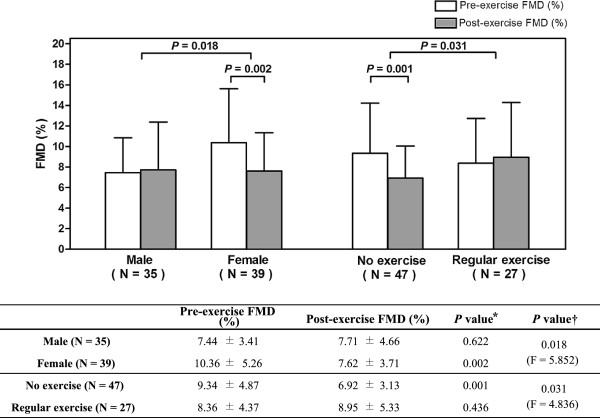
**Difference of FMD between pre-exercise and post-exercise according to gender and exercise habit.***P* values were calculated by RM-ANCOVA and the covariance was baseline brachial artery diameter before exercise. Reduction of FMD after acute exercise was observed in female subjects and those without regular exercise. * *P* values indicate the difference in each group. † *P* values indicate the overall difference. FMD = flow-mediated dilatation; RM-ANCOVA = repeated measure analysis of covariance.

To investigate the effects of gender and exercise habit on the change of FMD before and after exercise, repeated measure analysis of covariance (RM-ANCOVA) method was utilized. The differences of FMD after exercise and the influence of other factors were estimated, using baseline brachial artery diameter at rest as covariance (Figure 5). FMD was significantly reduced after exercise in female group (*P* = 0.002) and the change of FMD according to gender was significant (F = 5.852; *P* = 0.018). In subjects who did not exercise 3 or more days a week, post-exercise FMD was significantly impaired (*P* <0.001) and the change of FMD according to exercise habit was also significant (F = 4.836; *P* = 0.031).

**Figure 6:**
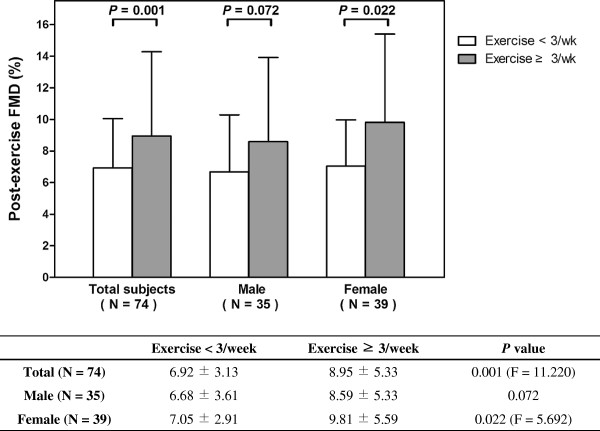
**Difference of post-exercise FMD according to exercise habit.***P* values were calculated by ANCOVA and the covariance was baseline brachial artery diameter before exercise. Post-exercise FMD showed significant difference according to exercise habit. In female group, subjects without regular exercise had impaired post-exercise FMD. ANCOVA = analysis of covariance; FMD = flow-mediated dilatation.

**Figure 7:**
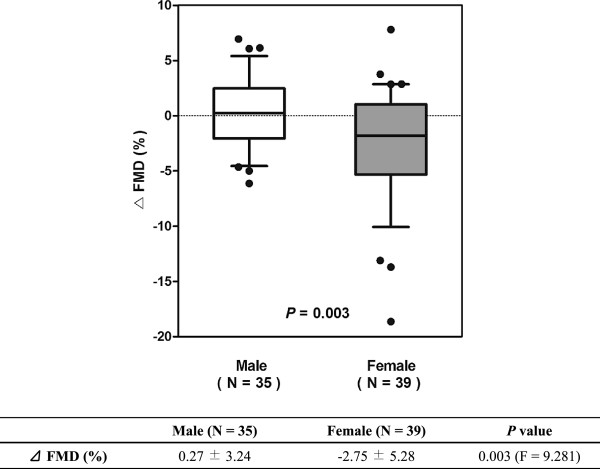
**Difference of ΔFMD according to gender.** The lower and upper ends of box indicate the quartiles at 25% and 75%, and the lower and upper bars indicate the whiskers at 10% and 90%, respectively. *P* value was calculated by ANCOVA and the covariance was baseline brachial artery diameter before exercise. The reduction of FMD after acute exercise was more prominent in female subjects. ANCOVA = analysis of covariance; FMD = flow-mediated dilatation.

Figure 6 shows the effects of gender and exercise habit on post-exercise FMD. For this analysis, ANCOVA method was used and the covariance was baseline brachial artery diameter. Subjects who exercise 3 or more days a week showed higher post-exercise FMD (F = 11.220; *P* = 0.001) and the significant difference was only found in female group (F = 5.692; *P* = 0.022). Also, ΔFMD was greater in female group (F = 9.281; *P* = 0.003) (Figure 7).

## Discussion

The present study investigated the impact of acute exercise on FMD and the influences of gender and exercise habit, in 74 young healthy subjects from society. In the present study, we found that FMD was reduced after exercise, and the reduction was observed in female group and subjects who do not exercise 3 or more days a week. There were significant differences according to gender and exercise habit, regarding the impact of acute exercise on FMD.

### Impact of exercise on FMD

FMD can be improved by exercise training, which is known for beneficial for cardiovascular diseases [17,18,21,28]. Although there have been lots of evidence implying the beneficial effect of exercise on endothelial function, the impact of acute exercise on FMD is still inconclusive. Silvestro et al. investigated the preventive effect of vitamin C on endothelial function, and they reported the reduced FMD after exercise in patients with intermittent claudication [23]. Highly endurance-trained male athletes showed reduced FMD 1 hour after intensive exercise in a study of Rognmo et al. [24]. Jones et al. reported decrease of FMD after exercise, in a study investigated the diurnal variation of FMD [22]. In contrast to these reports, sustained increase of FMD after acute exercise was observed in postmenopausal women [25]. Regarding healthy controls, Cosio-Lima et al. showed acute vasodilatory response after 30-minute walk, comparing FMD between healthy controls and kidney transplant recipients [26]. In a recent study of Llewellyn et al., some interesting findings were reported [29]. They found that endothelial function was reduced post-exercise when expressed as percent change in diameter, but the decrease of FMD was attenuated when it was normalized to shear rate (SR).

In the present study, post-exercise FMD was significantly reduced compared with pre-exercise (Figure 4). This result should be interpreted cautiously, because FMD is directly affected by the baseline brachial artery diameter. Exercise induces increase in blood flow and the augmented blood flow causes vasodilation, which directly impacts the magnitude of FMD [30]. For this reason, there have been efforts to normalize FMD according to SR. *Pyke et al.* and *Harris et al.* suggested a normalization method for SR [30-32], and it has been accepted as one of the appropriate methods for interpretation of FMD. However, FMD and SR after acute exercise showed weak relationship, suggesting this normalization method should not be applied for post-exercise data [29]. Instead, we adopted ANCOVA and RM-ANCOVA methods to normalize FMD using the pre-exercise baseline brachial artery diameter as covariance. Figure 2 shows the close correlation of pre-exercise baseline diameters with post-exercise diameters, pre- and post-exercise FMD. These results provide reasonable evidence for using baseline brachial artery diameter to normalize FMD.

Since the burden of acute exercise could affect the degree of vasodilation [25,27], and it might have influenced the results of this study, we also analyzed the relationship of exercise duration and METS with FMD (Figure 3). However, the exercise parameters were not associated with FMD measurements, indicating that the burden of exercise in this study did not influence the results.

The reduction of FMD after exercise mainly came from female group and subjects who do not exercise 3 or more days a week. Female group and subjects without regular exercise showed significant reduction of FMD (Figure 5) and ΔFMD was larger in female group (Figure 7). These results might be due to the difference of exercise habit between genders, as most of the subjects with regular exercise were male (Table 1). Nevertheless, ΔFMD showed significant correlation with exercise habit (β = 2.532; *P* = 0.027) even the effect of gender was adjusted (Table 3), suggesting the exercise habit is an important factor. The result shown in Figure 6 is another supporting evidence that exercise habit influences post-exercise FMD.

### Gender difference in FMD

In the present study, changes of FMD after exercise showed significant gender difference, which is worthy of attention. We should consider the reasons why women had higher pre-exercise FMD and why they had a greater FMD reduction after exercise.

Firstly, pre-exercise FMD was significantly higher in female group. This phenomenon was reported by a series of studies, showing similar results. In a study by *Jensen-Urstad and Johansson*, FMD was 3.1 ± 2.7% in male subjects and 5.7 ± 3.5% in female subjects at 35 years of age [33]. It could be inferred that smaller brachial artery diameter and hormonal status have influenced the results, since the smaller vessel shows the greater vasodilator response [34], and estrogen replacement therapy improves vascular function [35]. In a study of 2109 healthy adults aged 24 to 39 years by Juonala et al., male gender was an independent negative determinant of FMD [36]. Another important study by Mizia-Stec et al. provided an explanation regarding the difference in FMD between male and female patients with coronary artery disease [37]. In that study, FMD was significantly higher in female patients (12.9 ± 6.7 versus 8.9 ± 5.9%; P = 0.034), whereas baseline brachial artery diameter was higher in male patients (4.41 ± 0.6 versus 3.79 ± 0.5 mm; P = 0.012). Since the authors found that FMD was inversely correlated with baseline diameter, they arbitrarily compared the indices of “FMD × baseline diameter”, which were not different between male and female. These results are concordant with those of our study. Based on these evidences from previous studies, we could suggest that higher pre-exercise FMD in female group was come from the difference in baseline artery diameter. It might account for baseline gender difference in endogenous vasodilation and also difference in hormonal status.

Secondly, female subjects showed a greater decrease of FMD after exercise. In the present study, baseline brachial artery diameter was smaller in female group, while the increase of brachial artery diameter after exercise was larger in female. Compared to the baseline diameter, the increment was 4.2% in male and 6.8% in female. We believe that this might have resulted in the greater decrease of FMD after exercise in female group. Additionally, we could suggest that baseline endogenous vasodilation is different between male and female, but this difference is abolished after acute exercise, since vessel diameter is increased to maximum by exercise.

Importantly, we used baseline brachial artery diameter as covariance for ANCOVA or RM-ANCOVA tests. Thus, gender differences and also influences of exercise habit in our study are fairly independent from the influence of baseline diameter. We believe that we could show the important characteristics of acute exercise model of FMD, regardless of baseline diameter.

### Clinical implications

The results of the present study have some important points. First, regarding the interpretation of FMD, it should be tailored depending on the gender. Several previous studies and the present study commonly indicate that the impact of exercise on FMD is definitely different according to gender [16,17,38]. It might reflect the low overall physical activity level in female, which eventually impairs the endothelial capacity. Also it should be considered when FMD was used as a surrogate marker of the efficacy of clinical intervention. Second, exercise habit is an important factor determining the response of FMD to acute exercise. It suggests the beneficial effect of exercise on endothelial function, in concordance with previous studies [17,18,21,28]. In subjects who do not exercise regularly, vascular capacity might be impaired, and therefore, decreasing post-exercise FMD. Third, endothelial dysfunction could be manifested in healthy young adults as a form of impaired post-exercise FMD. Endothelial dysfunction is an early sign of atherosclerosis, and there have been several reports suggesting that endothelial dysfunction is present even in children with risk factors [5].

### Limitations

There are several limitations regarding this study. First, FMD was analyzed by calipers without a system for the automatic evaluation. Since the automatic measurement is more reproducible and less subjective [39,40], we should consider the probable bias from manual measurement. Despite the methodological limitation, our study showed evident results. It might be due to the experienced sonographers and rigorously standardized procedure [41]. Second, detailed assessment of exercise capacity was not done. The qualitative analysis of exercise capacity requires more specific information such as peak oxygen consumption (peak VO_2_) or ventilator efficiency (VE/VCO_2_), assessed by cardiopulmonary exercise test [42]. However, any study participants who could not reach at least 85% of age-predicted maximum heart rate in treadmill test were excluded on account of inadequate exercise loads in this study. It is reasonable to assume that this protocol enabled to estimate the impact of acute exercise with adequate load on FMD. Third, study participants were young healthy people. FMD responses to acute exercise in subjects with established cardiovascular risk factors or cardiovascular diseases need to be clarified. Fourth, all of our study participants were Korean ethnicity. There have been several reports suggesting ethnic differences between Asians and Caucasian, however, FMD of healthy young adults was similar in both ethnic groups [15,43,44].

## Conclusion

The present study showed that FMD was reduced after a bout of acute exercise in healthy young subjects. The impact of acute exercise showed significant differences according to gender and exercise habit. FMD impairment after acute exercise was observed in females and subjects without regular exercise. Our findings provide important information of FMD in the acute exercise model. Also, these support the gender differences in endothelial dysfunction and the importance of exercise habit.

## Abbreviations

ANCOVA: Analysis of covariance; BMI: Body mass index; CAD: Coronary artery disease; CI: Confidence interval; DM: Diabetes mellitus; FMD: Flow-mediated dilatation; HDL-C: High-density lipoprotein-cholesterol; IQR: Interquartile range; LDL-C: Low-density lipoprotein-cholesterol; NO: Nitric oxide; SD: Standard deviation; RM-ANCOVA: Repeated measure analysis of covariance.

## Competing interests

The authors declare that they have no competing interests.

## Authors’ contributions

JZ conceived of and designed the study. DS and MK made substantial contributions to conception and design. SK and YK contributed to data interpretation and revision of the manuscript. WC, MI and HK analyzed data and contributed to discussion of the results. KK collected and analyzed the data, searched for articles, analyzed the data and made substantial contribution to revision of the manuscript. IH collected, managed and analyzed the data, searched for articles, drafted the figures, and drafted the first manuscript. All authors read and approved the final manuscript.

## Funding

This study was supported by the National Research Foundation of Korea (NRF) grant funded by the Korea government (MEST) (2010–0021554, 2011–0025796).
